# Ascertaining breast cancer patient experiences through a journey map: A qualitative study protocol

**DOI:** 10.1371/journal.pone.0244355

**Published:** 2020-12-21

**Authors:** Laura Ciria-Suarez, Paula Jiménez-Fonseca, María Palacín-Lois, Mónica Antoñanzas-Basa, Ana Férnández-Montes, Aranzazu Manzano-Fernández, Beatriz Castelo, Elena Asensio-Martínez, Susana Hernando-Polo, Caterina Calderon

**Affiliations:** 1 Clinical Psychology and Psychobiology Department, Faculty of Psychology, University of Barcelona, Barcelona, Spain; 2 Medical Oncology Department, Hospital Universitario Central of Asturias, Oviedo, Spain; 3 Social Psychology and Quantitative Psychology Department, Faculty of Psychology, University of Barcelona, Barcelona, Spain; 4 Medical Oncology Department, Hospital Universitario Clínico San Carlos, Madrid, Spain; 5 Medical Oncology Department, Complejo Hospitalario Universitario de Orense, Orense, Spain; 6 Medical Oncology Department, Hospital Universitario La Paz, Madrid, Spain; 7 Medical Oncology Department, Hospital General Universitario de Elche, Elche, Spain; 8 Medical Oncology Department, Hospital Universitario Fundación Alcorcón, Madrid, Spain; Kaiser Permanente Washington, UNITED STATES

## Abstract

**Background:**

The current cancer care system must be improved if we are to have in-depth knowledge about breast cancer patients’ experiences throughout all the stages of their disease.

**Aim:**

This study seeks to describe breast cancer patients’ experience over the course of the various stages of illness by means of a journey model.

**Methods:**

This is a qualitative descriptive study. Individual, semi-structured interviews will be administered to women with breast cancer and breast cancer survivors. Patients will be recruited from nine large hospitals in Spain and intentional sampling will be used. Data will be collected by means of a semi-structured interview that was elaborated with the help of medical oncologists, nurses, and psycho-oncologists. Data will be processed adopting a thematic analysis approach.

**Discussion:**

The outcomes of this study will afford new insights into breast cancer patients’ experiences, providing guidance to improve the care given to these individuals. This protocol aims to describe the journey of patients with breast cancer through the healthcare system to establish baseline data that will serve as the basis for the development and implementation of a patient-centered, evidence-based clinical pathway.

## Introduction

The number of cases of cancer continues to grow around the world, thanks to populational screening and the progress and extension of diagnostic testing, as well as to the increase in the risk factors we are currently exposed to, in particular, aging [1]. Breast cancer (BC) is currently the second most common cancer overall and the leading cancer in women, with more than 2 million new cases diagnosed in 2018 [2]. It is estimated that in Spain, the year 2020 will witness almost 33 thousand new cases in women [3]. Nevertheless, due to therapeutic advances and early diagnosis, survival and mortality have improved in recent years [4].

Breast cancer patients tend to be diagnosed when they are relatively young, around 55 years of age [5]. These individuals, given treatment side effects and sequelae, are three times more likely to experience physical symptoms and psychological disorders than patients with other kinds of tumors [6, 7]. Once diagnosed, they go through different phases. If we are to support and accompany them in this process, we must have a profound understanding of their experiences. As put forth by Hall et al. [8], “patient voice” is a term that has become more common in healthcare contexts and serves to describe the whole of the patient’s experiences, encompassing thoughts, emotions, feelings, worries, and concerns, amongst others. Thus, we can ascertain how cancer affects patients—physically, emotionally, cognitively, socially, and spiritually. This “patient voice” can be depicted as a journey, an experiential map of the different stages of disease subjects go through [9]. Like all cancers, the person receives a diagnosis, prognosis, and treatment; they recover, and, in the event of relapse, develop metastases. This care continuum is what is known as a “cancer journey”.

To a greater or lesser extent, each cancer diagnosis conceals the story of a life or existential crisis [10] that necessitates a major effort to adjust and adapt that will entail psychological repercussions for the individual [11]. This diagnosis is generally unexpected and psychological distress ensues in practically all patients, including feelings of uncertainty, disbelief, hopelessness, vulnerability, anger, fear, anxiety, and sadness [12, 13]. All of this implies a drastic change that, on occasion, compels the person to assume a new identity as a cancer patient who must also confront employment, economic, and psychosocial effects. Their lifestyle and self-perception change and their families are also affected [10]. The most common physical and psychological symptoms in patients during and following breast cancer treatment include fatigue, pain, and depression [14–17], in addition to fear of relapse [18] and of treatment side effects [19]. These symptoms undermine their psychological wellbeing and quality of life after diagnosis, when initiating therapy, and following surgery or systemic treatment [15].

Qualitative studies are gaining relevance to better comprehend specific aspects, such as willingness (or not) to participate in these studies [20]; breast cancer symptom recognition and evaluation [21, 22]; the importance of social support [23] or physical exercise [24] on survival and quality of life of these individuals. Nevertheless, to the best of our knowledge, there are no studies that analyze patients’ experience from diagnosis to treatment and follow up. Therefore, the aim of this study is to portray breast cancer patients’ experiences as they travel through all the stages of the disease. Although the patient's role in the course of the disease and in medical decision making is increasingly important, little research has focused on the patient´s experiences [25, 26].

A breast cancer patient journey map will enable health care professionals to gain first-hand knowledge about their patients’ personal experiences, enhance communication and understanding in the physician-patient relationship, thereby creating a better, more person-centered system. Moreover, the journey information may aid in redesigning the service, improving quality, planning changes more effectively, and in shifting the focus of care toward activities most valued by the patient [27]. We hope that this protocol will encourage researchers to create more patient journey maps.

## Methods

### Study design

In this study, a qualitative method will be used to explore the pathway of standard care for women with breast cancer and to develop a schematic map of their journey process based on their experiences [27]. As Sandelowski comments [28], by means of this approach, the experience is detailed from the person’s perspective and, following analysis, an in-depth description of patient experiences is presented. Healthcare Process Mapping is an important new form of clinical audit that examines how we organize the patient´s journey, using the patient’s perspective to identify problems and suggest improvements [29]. It allows us to understand the patient’s experience by separating the management of a specific condition or treatment into a series of consecutive events or steps (e.g., diagnostic procedures, therapeutic interventions, staff interactions, activities). Process Mapping has shown clinical benefit across a variety of specialties, multidisciplinary teams, and healthcare systems [27].

This study will be performed in accordance with the ethical standards of the Declaration of Helsinki and its later amendments. This study was approved by the Research Ethics Committee of University of Barcelona (Institutional Review Board: IRB00003099) and supported by the Bioethic Group of the Spanish Society of Medical Oncology (SEOM) 2018 grant.

The study will be conducted in nine large hospitals in six geographical areas in Spain. Each study site has more than 100 beds and reference and tertiary oncology services.

### Participants

The presence of women with breast cancer and breast cancer survivors with a team of medical oncologists, oncology nurses and psycho-oncologists as consultants is anticipated. Study participants will comprise women with breast cancer who go to the sites for follow-up between December 2019 and January 2021. Inclusion criteria will be having been diagnosed with a histologically confirmed adenocarcinoma of the breast in the last 5 years and being over the age of 18 years. Those who are not in good physical/ mental state during the study period will be excluded. Medical oncologists and nurses who work at the centers will help to identify patients who meet the inclusion criteria, develop the written material, and interpret and clinically contextualize the results of the interview, but will not actively participate in the interviews.

An intentional sampling procedure will be used to recruit participants for the study based on age, marital status, level of education, employment status, having children, as well as clinical data, such as the stage of the disease and type of cancer treatment received. Therefore, patients will be interviewed with different cancer stages and who find themselves at different moments in their journey. The aim is to access the various experiences and promote transferability of the findings. Participation will be voluntary, and all participants will be asked about their own experiences.

### Data collection

A bibliographic search will be conducted on Medline and Scopus Electronic Database for the terms “breast cancer”, “wellbeing”, “mental health”, and “patient voice”, since 2015, with the aim of delving into the theoretical and conceptual framework of the topic. Based on this review, an interview guide will be created for data collection. A pilot test will be conducted in a public university hospital to evaluate the acceptability and ease of the interview process. The interview guide will be reviewed by two oncologists, three nurses (a day hospital cancer nurse, a case manager nurse who liaises with the different services, and the nurse in charge of explaining postoperative care and treatment), as well as two psycho-oncologists to make the necessary modifications prior to conducting the final interviews included in the analysis (S1 File). The interview will cover four main blocks. First, patients’ sociodemographic and medical information will be gathered. Second, daily activities, family, and support network will be discussed. Third, participants will be asked about their overall perception of breast cancer and their coping mechanisms. Finally, physical, emotional, cognitive, spiritual, and medical aspects related to diagnosis, treatment, and side effects will be probed. Additionally, patients will be encouraged to express their thoughts should they have more to say about the subject.

All the interviews will be performed by the fist author of the manuscript in a private space on the hospital grounds; if that is not possible, it will be conducted online. All the interviews will be recorded with the consent of the study subjects. Relevant notes will also be taken during the interview to document key issues and observations. The interviews will continue until the point of relative saturation is reached as regards the issues being discussed, lasting between 60 and 75 minutes. In general, a single interview will be carried out, although the contact will be saved, in case clarification is need for any aspect of the interview that had not been clearly recorded. To avoid bias arising from the timing of the interview, patients are three different time points will be represented: 1) after surgery, 2) during systemic treatment, and 3) at some point during the year following treatment completion.

### Data analysis

The data will undergo a qualitative content analysis. To assure trustworthiness, the analysis will be based on the system put forth by Graneheim and Lundman [30]. First, transcribing the interviews for analysis and reading them over several times so as to be familiar with the material and gain a broad understanding of the experiences of patients with breast cancer. This will be followed by a more deductive analysis of those aspects of patient´s experiences that are of primary interest to the research team (i.e., knowledge and experience of their cancer and treatment, risk awareness, experiences of cancer care services, overall health status, satisfaction with cancer services, and perceived health care needs). Next, it will continue dividing the text into different content areas; obtaining units of meaning and putting them into each content area; extracting and adding a unit of meaning code; categorizing codes in terms of differences and similarities, and creating themes to link underlying meanings in the categories. Key aspects of cancer-patient´s knowledge and experience will be identified. A subject analysis approach will be used to categorize the codes by means of several iteration. This will be done by the authors of the manuscript. Team members will review the data and triangulate the outcomes between two sources of data: qualitative data from the interview and non-modifiable information, such as sociodemographic (i.e., age, marital status, having children) and clinical (i.e., cancer stage, and surgery type) data. The categories will be discussed at length and validated by all the authors to guarantee that the issues put forth are cogent.

### Informed consent materials

All participants will receive a written informed consent form that they will sign prior to commencing with the interviews and after receiving information about the study. Said consent form will guarantee that they have received clear and sufficient information about the study; that their decision is voluntary, and that they understand that they can withdraw at any time. Likewise, they consent to the collection, analysis, and release of the information they share, to recording of the interview, and to the use of their own words in future publications.

## Discussion

### Contribution

So far as we are aware, this study will be the first qualitative research that will describe the experience of patients with breast cancer throughout the various stages of the disease using a journey model.

The knowledge garnered will enable us to comprehend breast cancer patients’ experiences in detail, as well as the reasons for them. Furthermore, having first-person information from these patients will promote greater understanding of their situation, their emotional state, and the empathy of all the stakeholders, supporting sufferers and their families in receiving holistic cancer care. In addition, this information is subject to being used at a micro level (for instance, giving knowledge to healthcare professionals so that they can better understand and communicate with their patients, or shedding more light on key aspects of breast cancer patients’ experiences, such as the impact of diagnosis, questioning their own identity and femininity, their approach to family communication–especially between mother and children-, repercussions for their partner and in the workplace, their feelings and thoughts about the treatment, or the significance they attached to the disease and the personal changes it brings), as well as at a macro level (for example, redesigning care or integrating specific services provided by the healthcare system) to enhance these women’s experiences. In this way, the current paradigm that advocates for a patient-based healthcare system will continue to be fostered.

This protocol offers a clear methodological guide for patients’ journey in healthcare. Journey maps, which originate in the field of service design [31], have been used in various areas to illustrate complex processes or interactions that would otherwise be difficult to apprehend. In the field of healthcare, journey maps can be used to depict service from the perspective of different stakeholders, like the patient, giving them a central role [32]. Healthcare process mapping is considered a new and important form of clinical auditing that examines how the patient journey is managed [27, 33]. This protocol seeks to encourage the realization of Patient Journeys, a highly useful tool that is still uncommon in investigation, giving researchers greater confidence, as well as improving study reliability.

There is a clear need to prepare patients facing this diagnosis for prolonged, multimodal treatment (surgery, chemotherapy, hormonotherapy, radiotherapy) and to confront physical and psychological sequelae, in addition to the fear surrounding an uncertain prognosis. It’s important to manage their expectations; similarly, identifying and addressing the issues that arise at different time points during treatment for their disease could improve the patient’s experience.

### Study limitations

This study will be conducted with Spanish participants, which is why certain aspects cannot reflect the experiences of breast cancer patients from other countries, both because of the particularities of the Spanish healthcare system and Spanish culture. Likewise, the data attained will be specific to women with breast cancer, which can scarcely be extrapolated to individuals with other cancers. Finally, the findings do not reflect men's experiences with breast cancer and research with this group would enrich the field further.

## Supporting information

S1 File This is the semi-structured interview guide to breast cancer patients/survivors. (DOCX)
