# Supplementary material for: Ascertaining breast cancer patient experiences through a journey map: A qualitative study protocol
Source: PLoS One. 2020 Dec 21;15(12):e0244355. doi: 10.1371/journal.pone.0244355 (PMC7751958; doi:10.1371/journal.pone.0244355)
Supplement: S1 File — (DOCX) [file pone.0244355.s001.docx]

**S1 File: Semi-structured interview guide to breast cancer patients/survivals.**

**Introduction**

Thank you for agreeing to take participate in this interview. We are conducting these interviews with the objective of understanding the experiences that people who are diagnosed with breast cancer go through, and in this way to be able to improve their experiences. Please, note that there are no right or wrong answers; we are only interested in your thoughts and opinions. Participation in this study is voluntary and you may withdraw from the study at any time without explanation. The interview will take approximately one and a half to two hours. With your permission, I would like to record the interview because I don’t want to miss any of your comments. All answers will be confidential. Do you have any questions? May I start the interview? Thus, in order to protect your privacy, we are providing you with a **signed confidentiality agreement**. I would like you to read it carefully and sign it.

*Please note that this guide only represents the main topics to be discussed with the participants and that these will be adapted to the patient's own speech and circumstances.*

**Initials data**

1. Before starting with your experiences, I would like to ask you for sociodemographic and clinical data. Could you tell me...

- Date of birth
- Marital status
- Number of children
- Educational Level
- Current ocupational status
- Place of residence
- Year of breast cancer diagnosis
- Cancer stage
- Other relevant information about the type of cancer (location, histological subtype, hereditary...) that you know
- Health care coverage: Social Security or private insurance

**The patient and the breast cancer (BC) perception**

2. To begin with, I would like you to tell me a little bit about yourself, for example, about your day-to-day life, profession, hobbies, family, support network...

3. And before we go into your experiences, I would like you to describe to me in a generic way how you would describe breast cancer.

4. I would like you to imagine now that you walk into an art gallery and at the back of the room you see a painting. Imagine you walk up to it and the sign on the painting says breast cancer. What would this painting look like to you? Why? And if you had to give it a title, what would it be?

**Patient Journey Mapping**

5. We will now go over your entire history with breast cancer on a temporary basis. We are going to start with what your life was like before the diagnosis and we are going to gradually advance through the different stages: diagnosis, treatment alternatives, decision on surgery, complementary treatment with chemotherapy and radiotherapy, secondary effects, revisions...

*The patient is asked about each of her stages. Given that the circumstances are different for each of the patients and not all of them go through the same phases (i.e. some are subjected to neo-adjuvant chemotherapy, others to adjuvant chemotherapy, others to radiotherapy, others have hormonal treatment, others suffer a recurrence...), we detail below different aspects to be covered in the different stages, asking "could you give me more information about..." or "how did you live...".*

- Medical issues: diagnostic studies, treatments, relationship with referring professionals (i.e. gynecologists, oncologists, radiologists, nurses, surgeons), decisions regarding treatment or type of surgery, side effects, use of alternative and/or complementary treatments

- Physical issues: physical discomfort and pain, aesthetic changes, day-to-day affectation, early menopause, personal care

- Emotional issues: anxiety and depression, self-esteem, fear of recurrence, uncertainty of the future, fear of death, early mourning, difficulties and concerns (i.e. about motherhood), emotional support network

- Cognitive issues: previous knowledge about the disease and treatments, doubts, sources of information, construction of identity (i.e. after breast surgery, during chemotherapy with hair loss), concept of femininity, coping strategies

- Social issues: communication of the disease (i.e. to the mother, children or closed environment), relationship with the partner (i.e. sexuality), children and with the community, friendships, work situation, contact with other patients or associations.

- Needs: outstanding needs and possible solutions

6. As the last section of this journey map, I would like you to tell me, from each of the stages of the disease you have gone through, a word, phrase or feeling that summarizes it. I would ask you to tell me the first one that comes to mind.

**Conclusion**

7. Finally, I would like to ask you if you give any meaning to this whole process (i.e. impulse to change your life, have other values or priorities).

8. Do you think this process has transformed you in any way? If so, could you define for me what you were like before and what you are like now?

**Thank you very much for your time and the information you shared today.**
